# Pressed for Hard Facts: Multiple Tetrachloroethylene–Cancer Links Go Unconfirmed

**Published:** 2006-02

**Authors:** Harvey Black

Recent U.S. studies have linked dry cleaners’ exposure to tetra-chloroethylene, a solvent used in the industry, to an increased risk for a number of cancers, including esophageal, lung, and cervical cancer. Results of other studies on bladder and pancreatic cancer are equivocal. Still other studies have shown an increased risk of non-Hodgkin lymphoma. Now a study by a team of Nordic researchers of cancer risk among dry-cleaning workers in Denmark, Finland, Norway, and Sweden finds that, with the exception of bladder cancer, exposure to the solvent showed no link to the disease **[*EHP* 114:213–219]**.

The researchers identified 46,768 dry-cleaning and laundry workers from the 1970 censuses in the four countries. The investigation was a series of case–control studies nested within this cohort of workers. Controls were matched by country, sex, and five-year group for age and year of cancer diagnosis.

The team studied the period from 1964 to 1979, when tetrachloroethylene was the primary solvent used for dry cleaning in these countries. The team considered four categories of exposure: exposed workers in dry-cleaning shops with fewer than ten workers (reflecting a probability of sharing tasks and working in more cramped quarters), other workers in dry-cleaning shops (such as seamstresses and office workers), unexposed laundry workers and others not working in a dry-cleaning shop, and those who could not be classified.

Blinded telephone interviews were done with cases, controls, and, eventually, next of kin in Norway and Sweden. If the subject’s occupation was dry cleaning, the interview covered length of employment in the dry-cleaning shop, number of workers in the shop, solvents used, and the subject’s smoking and drinking habits. For Denmark and Finland, pension records and other data sources were used to gather comparable information.

Although exposure to tetrachloroethylene varied greatly among shops, the average annual level of exposure was fairly stable between 1964 and 1979. The team found no increase in risk of cancers of the esophagus, gastric cardia, liver, pancreas, or kidney. There was also no link to non-Hodgkin lymphoma. The study did, however, find a 44% excess risk in bladder cancer concentrated in Norway and Denmark, the two countries with the best data.

The authors point to several strengths of the study, particularly its completeness. It covered all persons working in dry cleaning in 1970 in the Nordic countries. It also compared two cohorts, dry-cleaning workers and laundry workers, who had similar jobs except for the exposure to tetrachloroethylene.

The authors also acknowledge the study had some weaknesses. For a high proportion of cases and controls from Sweden and Finland the authors could not determine whether the subjects worked in either a laundry or dry-cleaning business. Consequently, estimates of cancer risk were reported for all four countries together and for Denmark and Norway together. Furthermore, subjects could not be classified by exposure level to tetrachloroethylene. The researchers note, though, that because the data indicated a fairly stable level of exposure during employment, they consider length of time on the job an adequate surrogate measure of a cumulative dose.

## Figures and Tables

**Figure:**
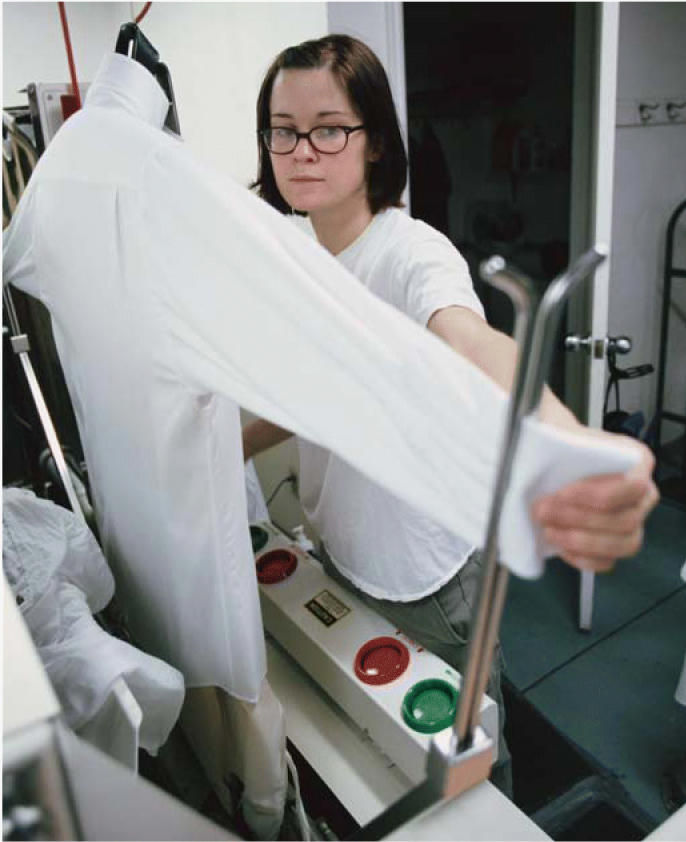
Clean sweep for dry cleaners? Tetrachloroethylene, a solvent widely used in the dry-cleaning industry, has been implicated in many cancers, but a new study of Nordic dry cleaners fails to corroborate most of those links.

